# Influenza Vaccine Effectiveness in the Netherlands from 2003/2004 through 2013/2014: The Importance of Circulating Influenza Virus Types and Subtypes

**DOI:** 10.1371/journal.pone.0169528

**Published:** 2017-01-09

**Authors:** Maryam Darvishian, Frederika Dijkstra, Eva van Doorn, Maarten J. Bijlsma, Gé A. Donker, Marit M. A. de Lange, Laura M. Cadenau, Eelko Hak, Adam Meijer

**Affiliations:** 1 Department of Epidemiology, University Medical Center Groningen, University of Groningen, Groningen, the Netherlands; 2 Unit of PharmacoEpidemiology & PharmacoEconomics (PE2), Department of Pharmacy, University of Groningen, Groningen, the Netherlands; 3 British Columbia Centre for Disease Control, Vancouver, BC, Canada; 4 School of Population and Public Health, University of British Columbia, Vancouver, BC, Canada; 5 Infectious Disease Epidemiology and Surveillance, Centre for Infectious Disease Control, National Institute for Public Health and the Environment (RIVM), Bilthoven, the Netherlands; 6 Sentinel Practices, NIVEL Primary Care Database, Utrecht, the Netherlands; 7 Infectious Disease Research, Diagnostics and Screening, Centre for Infectious Disease Control, National Institute for Public Health and the Environment (RIVM), Bilthoven, the Netherlands; Public Health Agency of Canada, CANADA

## Abstract

Influenza vaccine effectiveness (IVE) varies over different influenza seasons and virus (sub)types/lineages. To assess the association between IVE and circulating influenza virus (sub)types/lineages, we estimated the overall and (sub)type specific IVE in the Netherlands. We conducted a test-negative case control study among subjects with influenza-like illness or acute respiratory tract infection consulting the Sentinel Practices over 11 influenza seasons (2003/2004 through 2013/2014) in the Netherlands. The adjusted IVE was estimated using generalized linear mixed modelling and multiple logistic regression. In seven seasons vaccine strains did not match the circulating viruses. Overall adjusted IVE was 40% (95% CI 18 to 56%) and 20% (95% CI -5 to 38%) when vaccine (partially)matched and mismatched the circulating viruses, respectively. When A(H3N2) was the predominant virus, IVE was 38% (95% CI 14 to 55%). IVE against infection with former seasonal A(H1N1) virus was 83% (95% CI 52 to 94%), and with B virus 67% (95% CI 55 to 76%). In conclusion IVE estimates were particularly low when vaccine mismatched the circulating viruses and A(H3N2) was the predominant influenza virus subtype. Tremendous effort is required to improve vaccine production procedure and to explore the factors that influence the IVE against A(H3N2) virus.

## Introduction

Influenza viruses cause annual epidemics worldwide. Although most infections are relatively mild, in high-risk groups such as people aged 60 years and older the infection can lead to serious complications, hospital admission, and mortality [1]. In the last decades influenza epidemics were caused by type A (subtypes A(H1N1) through 2009, A(H1N1)pdm09 from 2009 onwards and A(H3N2)), and type B influenza viruses (two different genetic lineages: B/Victoria/2/87 (Victoria) and B/Yamagata/16/88 (Yamagata)) [1–2].

According to the World Health Organization (WHO), seasonal influenza vaccination is the main strategy to prevent influenza and its complications [1]. However, due to the changing nature of the influenza virus (antigenic drift), the vaccine composition and the influenza vaccine effectiveness (IVE) can vary each year [3]. To decide about the composition of the influenza vaccine for upcoming Southern or Northern hemisphere season, WHO organizes consultations with an advisory group of experts to analyze the influenza surveillance and the antigenic characteristics data of circulating viruses in order to issue recommendations. The annual recommendations for the Northern hemisphere and Southern hemisphere are given in February and September, respectively [4]. The national and supra-national vaccine regulatory agencies approve the vaccine composition and WHO essential regulatory laboratories and regional pharmaceutical companies then develop and produce the vaccine based on the WHO recommendation, which will take about half a year. Despite all the efforts, the antigenic match between influenza vaccine strains and circulating influenza viruses is sometimes suboptimal, which could consequently affect the IVE [5]. For instance, in influenza season 2014/15 the antigenic mismatch between the vaccine strain and the predominant virus subtype A(H3N2) resulted in a lower IVE and higher excess mortality rate in the Netherlands [6].

Additionally, since IVE can vary from year to year it is important to measure and monitor the IVE annually. It has been shown that even within a same country, IVE estimates have a high fluctuation over different influenza seasons [7–11]. For example, the currently available overall IVE estimates for the Netherlands range from 59% in season 2007/08 to 5% in season 2011/12 [12–13]. Moreover, there are indications that IVE differs per virus type and subtype/lineage [10–11]. In a study conducted in Denmark, IVE for influenza A was −11%; whereas the IVE for influenza B was point estimated at 69% during influenza season 2012/13 [11].

To gain more insight in a possible association between IVE and circulating influenza virus (sub)types/lineages, we conducted a test-negative design case-control study to estimate the overall and (sub)type/lineage specific IVE in preventing laboratory-confirmed influenza in the Netherlands over a series of 11 seasons. Additionally, we explored the relationship between the estimated IVEs with the dominancy of virus (sub)types/lineages and level of vaccine match during these influenza seasons.

## Methods

### Study design and population

The Sentinel Practices of NIVEL Primary Care Database cover about 0·7% of the Dutch population and being nationally representative by age, population density and regional distribution [14–15]. Each participating sentinel general practitioner (GP) is asked to take a throat swab and nose swab from two patients with influenza-like illness (ILI) symptoms on a weekly basis. If no ILI patient in a week is encountered the GP is asked to swab patients with another acute respiratory tract infection (ARI) [16]. ILI is defined according to the ‘Pel’ criteria [17] as an acute onset of symptoms (prodromal stage ≤ 4 days) including a rectal temperature of at least 38 degrees Celsius and at least one respiratory or systemic symptom (i.e. cough, nasal catarrh, sore throat, frontal headache, retrosternal pain, myalgia). ARI is defined as an acute respiratory illness other than ILI, such as acute sinusitis or pneumonia, and with at least one of the following symptoms; coughing, rhinorrhea or sore throat [18]. Both ILI and ARI patients were included in this study to maximize the power. As required by Dutch legislation, the influenza surveillance is registered in the Personal Data Protection Act Register of the Personal Data Protection Commission (RIVM/EPI-043). No further ethical approval was needed for the IVE study, because only anonymised data was used.

### Covariates

Data on potential confounding and effect modifiers were collected which included information on date of birth (recoded as age groups 0–4, 5–14, 15–59 and ≥ 60 years at swabbing date for the IVE study dataset), gender, date of symptom onset and swabbing (recoded as delay between both dates as <3 days, 3–5 days and 6–7 days), seasonal influenza vaccination status, respiratory allergy, patient diagnosis (ILI and ARI), influenza season and occurrence of underlying medical conditions (diabetes, cardiovascular disease, chronic pulmonary disease and immunodeficiency).

### Study design and outcome

In order to estimate IVE against specific outcome, laboratory confirmed influenza, and partially adjust for differences in health care-seeking behaviour of cases and controls, we performed a test-negative design case-control study [19]. Cases were ILI or ARI patients who tested positive for at least one of the influenza virus subtypes: former seasonal A(H1N1), A(H1N1)pdm09, A(H3N2), or influenza B lineages Victoria and Yamagata, and controls were ILI or ARI patients who tested negative for all the above mentioned virus subtypes/lineages. Both ILI and ARI patients swabbed in the seasons 2003/04 through 2013/14 were included in the analyses. Only patients that were swabbed within the period that swabs tested positive for influenza virus (i.e. influenza virus was circulating) were selected as cases or controls (Table 1). Patients were excluded if (1) the vaccination status was missing; (2) the swabs were collected more than 7 days after symptom onset (since the viral load of the specimens collected 7 days after onset of ILI in general has dropped below the detection limit of the diagnostic test and therefore estimation of the subject status is not reliable); (3) the date of swabbing was before the first of December of each season (since the vaccine campaign in the Netherlands is from half October to half November; the inclusion date was set to 1 December to make sure that vaccination was given at least 14 days before symptom onset); (4) the patient used antiviral medication within the two weeks prior to swabbing (this was done because antiviral medication can influence the viral load and consequently the chance of virus detection); (5) data on ILI and ARI diagnosis, age, or underlying chronic illness was missing [20, 21].

**Table 1:** Baseline characteristics of laboratory-confirmed influenza cases and test-negative controls, seasons 2003/04 through 2013/14, Netherlands.

**Variable **	**Cases (Number/Total (%))**	**Controls (Number/Total (%))**	*P*-Value*
**Seasonal vaccination received**	197/1422 (14·8)	754/3410 (22·1)	<0·001
**03/04**	6/34 (17·6)	21/79 (26·6)	
**04/05**	22/73 (30·1)	37/149 (24·8)	
**05/06**	14/110 (12·7)	44/278 (15·8)	
**06/07**	10/94 (10·6)	73/309 (23·6)	
**07/08**	13/191 (6·8)	64/417 (15·3)	
**08/09**	21/197 (10·7)	78/461 (16·9)	
**09/10**	8/33 (24·2)	92/289 (31·8)	
**10/11**	27/246 (11·0)	81/346 (23·4)	
**11/12**	15/81 (18·5)	91/434 (21·0)	
**12/13**	53/311 (17·0)	82/332 (24·7)	
**13/14**	8/52 (15·4)	91/316 (28·8)	
**Gender^a^**			0·089
**Male**	683/1412 (48·4)	1544/3380 (45·7)	
**Female**	729/1412 (51·6)	1836/3380 (54·3)	
**Age group**			<0·001
**0–4 years**	128/1422 (9·0)	503/3410(14·8)	
**5–14 years**	263/1422 (18·5)	364/3410 (10·7)	
**15–59 years**	886/1422 (62·3)	2009/3410 (58·9)	
**≥ 60 years**	145/1422 (10·2)	534/3410 (15·7)	
**Diagnosis**			<0·.001
**ILI**	1142/1422 (80·3)	1827/3410 (53·6)	
**ARI**	280/1422 (19·7)	1583/3410 (46·4)	
**Interval from symptom onset to swab date, days**			<0·001
**<3 d**	523/1422 (36·8)	1193/3410 (35·0)	
**3–5 d**	776/1422 (54·6)	1743/3410 (51·1)	
**>5 d**	123/1422 (8·6)	474/3410 (13·9)	
**Chronic condition by age**	95/1422 (6·7)	380/3410 (11·1)	<0·001
**0–4 years**	3/95 (3·2)	14/380 (3·7)	
**5–14 years**	5/95 (5·3)	23/380 (6·0)	
**15–59 years**	56/95 (58·9)	182/380 (47·9)	
**≥ 60 years**	31/95 (32·6)	161/380 (42·4)	
**Respiratory allerg by age^b^**	114/1422 (8·0)	248/3410 (7·3)	0·374
**0–4 years**	5/114 (4·4)	15/248 (6·1)	
**5–14 years**	25/114 (21·9)	38/248 (15·3)	
**15–59 years**	72/1114 (63·2)	159/248 (64·1)	
**≥ 60 years**	12/114 (10·5)	36/248 (14·5)	
**Seasonal vaccination by age**	198/1430(13·8)	883/3410 (22·1)	<0·001
**0–4 years**	5/128 (3·9)	41/566 (7·2)	
**5–14 years**	13/264 (4·9)	28/403 (6·9)	
**15–59 years**	96/892 (10·8)	357/2290 (15·6)	
**≥ 60 years**	84/146 (57·5)	457/622 (73·5)	

*Statistically significant at P < 0·05

^a^ Number of missing = 40

^b^Number of missing = 50

The beginning of the ‘positive swab test period’ was determined for each season separately and started if two subsequent weeks had positive test results with the ILI incidence exceeding the baseline activity threshold (51/100,000 population) at national level. The end of the ‘positive swab test period’ was marked when two subsequent weeks had zero positive test results for influenza virus. The influenza vaccine was considered matched with circulating viruses if at least one of the two following criteria was fulfilled: (1) all the vaccine components were antigenically similar to the circulating A subtypes (H1N1 or H1N1pdm09 and H3N2) and B lineages (Victoria or Yamagata); (2) vaccine strain antigenically matched the predominant and one of the non-predominant circulating virus subtypes. An influenza virus type or subtype/lineage was considered as predominant when detected in a proportion of ≥60% among the total influenza virus detections in week 40 through week 39 of the following year [22]. The influenza vaccine was considered to partially matched if: (1) vaccine strain matched the predominant virus subtype but mismatched the non-predominant subtypes. In all other situations the vaccine was considered mismatched with circulating viruses. Antigenic match data, derived using ferret sera raised against vaccine reference strains, were extracted from data published by the Dutch National Influenza Centre [23–33].

### Laboratory analysis

The collected swabs were sent to the National Institute for Public Health and the Environment (RIVM) for diagnostic testing and virus subtype/lineage determination when positive for influenza virus [14–15]. Reverse transcription polymerase chain reaction (RT-PCR) was used for influenza virus detection throughout the study period. Influenza virus positive specimens were further subtyped (type A viruses by hemagglutination inhibition assay (HI) or RT-PCR during seasons 2003/2004 through 2007/2008 and since the 2008/2009 season by RT-PCR only) or the lineage determined (type B viruses by HI; since season 2010/2011 by RT-PCR).

### Statistical analysis

The descriptive and clinical characteristics of cases and controls were compared by using Chi-square test and P-value < 0.05 was considered statistically significant. To assess IVE for each influenza season separately, multiple logistic regression model was used. IVE was estimated by using the formula (1 –odds ratio (OR)) × 100%, where OR is the ratio between the odds of vaccination between influenza test-positive cases and influenza test-negative controls.

To assess the overall IVE over the 11 influenza seasons we used generalized linear mixed-effect model (GLMM) with logit link, in which influenza seasons are modelled as a random effect. Moreover, GLMM was used to estimate the overall IVE stratified by influenza virus (sub)types and vaccine match status (Table 1) (S1 File).

To adjust for confounders, the variables that changed the crude OR by more than 5% were included in the GLMM or multiple logistic regression model (S1 File).

The adjusted IVE were calculated for each influenza season separately. In case of low statistical power in the stratified analysis, only unadjusted IVE was estimated. IVE was considered significant when the 95% confidence interval (95% CI) did not contain zero or negative value. Statistical analyses were conducted using SAS software (version 9.4) and SPSS software (IBM Corp. Released 2013. IBM SPSS Statistics for Windows, Version 22.0. Armonk, NY: IBM Corp).

## Results

### Patient characteristics

From the total of 11,199 subjects who were swabbed from 2003/2004 through 2013/2014 influenza seasons, 4,832 (43·15%) ILI and ARI patients met the inclusion criteria (Table 1). A total of 1422 (29%) patients tested positive (cases) and 3410 (71%) tested negative (controls) for influenza virus. Cases and controls were similar with respect to gender and presence of respiratory allergy. However, compared with control subjects, cases presented more frequently with ILI symptoms (80% vs 54%), were more within the age groups of 5–14 years (18% vs 11%) and 15–59 years (62% vs 59%), were less vaccinated (14% vs 22%), and had a lower incidence of chronic medical illness (7% vs 11%) (Table 1).

### Overall influenza vaccine effectiveness

Among all the potential confounders, age, chronic medical illness, and influenza season were the only variables that changed the crude OR by more than 5% and therefore were included in the GLMM estimating IVE over all influenza seasons or multiple logistic regressions estimating overall IVE for each influenza season (overall estimate not stratified by subtype).

**Fig 1:**
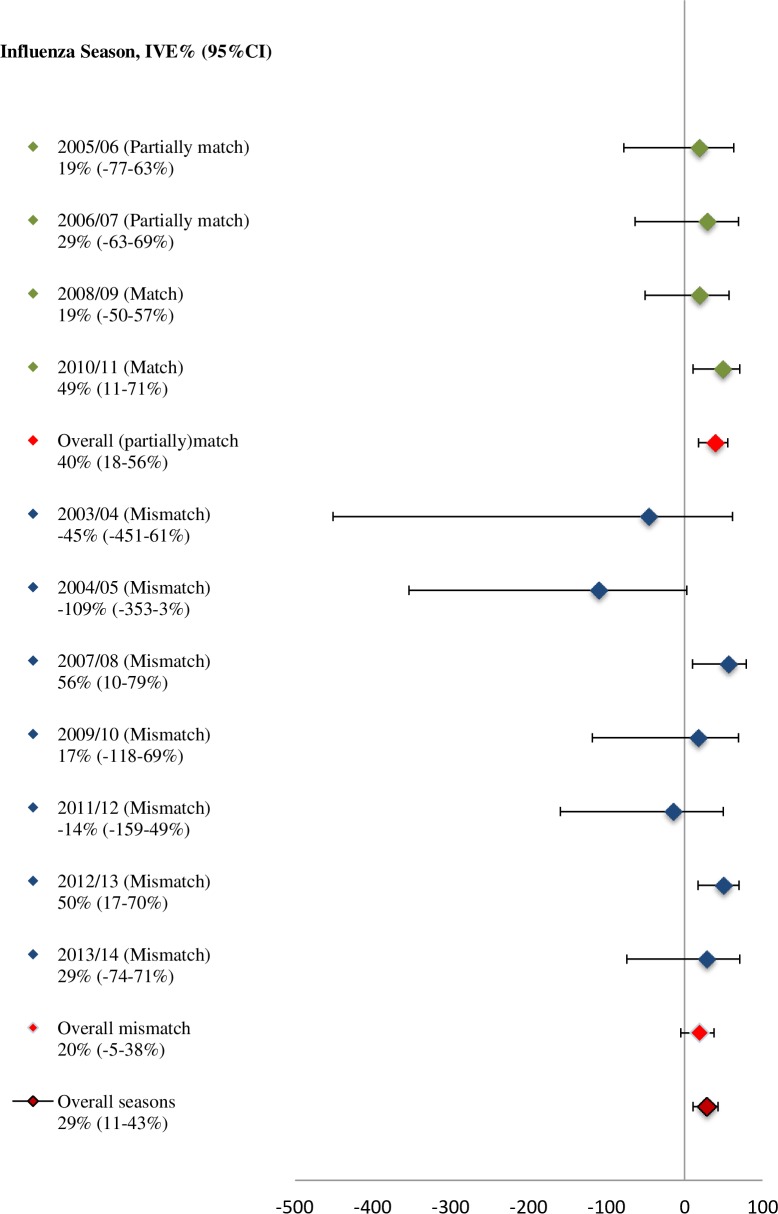
Overall adjusted IVE (%) (adjusted for age, chronic medical illness and influenza seasons), and its 95% CI for the (partially)matched and mismatched influenza seasons 2003/04-2013/14.

Using GLMM, the overall IVE adjusted for age, chronic medical illness and influenza season was 29% (95% CI 11 to 43%) (Fig 1). Furthermore, the overall adjusted IVE was 40% (95% CI 18 to 56%) when vaccine (partially)matched (influenza seasons 2005/06, 2006/07, 2008/09, and 2010/11) and 20% (95% CI -5 to 38%) when vaccine did not match the circulating viruses (influenza seasons 2003/04, 2004/05, 2007/08, 2009/10, 2011/12, 2012/13, and 2013/14).

Using multiple logistic regression for each influenza season, vaccine showed statistically significant effectiveness against laboratory-confirmed influenza for 2010/11 influenza season when vaccine matched (IVE: 49%; 95% CI 11 to 71%) and for influenza seasons 2007/08 and 2012/13 when vaccine did not match the circulating viruses (IVE: 57%; 95% CI 10 to 79% and IVE: 50%; 95% CI 17 to 70%, respectively) (Fig 1).

### Influenza vaccine effectiveness by influenza virus type and subtype/lineage

#### Unadjusted IVE against influenza A(H3N2)

The overall IVE against influenza A(H3N2) (nine seasons) was 20% (95% CI -4 to 38%). Restricting the analysis to the influenza seasons when the A(H3N2) was the predominant virus (2003/04, 2006/07, 2008/09, 2011/12 and 2013/14), IVE increased to 38% (95% CI 14 to 55%). Moreover, IVE was statistically significant against influenza A(H3N2) for season 2006/07; 54% (95% CI 4 to 78%) (Table 2).

**Table 2:** Overall and (sub)type/lineage specific IVE and the 95% CI for influenza seasons 2003/04 through 2013/14, Netherlands.

	**Influenza virus (sub)types/lineages; IVE% (95% CI)**					
**Influenza Season**	Former A(H1N1)	A(H1N1)pdm09	A(H3N2)	B (total)	B Yamagata	B Victoria
**2003/04**	NE		54% (-48-86%)	NE		
**2004/05**	66% (-147-96%)		NE	57% (-53-88%)		
**2005/06**	NE		-6% (-171-58%)	34% (-65-73%)		
**2006/07**	NE		54% (4–78%)*	NE		
**2007/08**	81% (22–96%)*		-10% (-199-59%)	68% (17–88%)*		
**2008/09**	NE		31% (-199-60%)	78% (-16-94%)		
**2009/10**	NE	25% (-72-68%)	NE	NE		
**2010/11**	NE	43% (-3-68%)	NE	73% (47–87%)*	34% (-205-86%)	76% (49–89%)*
**2011/12**	NE	NE	11% (-70-53%)	6% (-351-82%)	NE	
**2012/13**	NE	51% (3–75%)*	-41% (-140-16%)	64% (44–79%)*	67% (40–82%)*	
**2013/14**	NE	72% (-12-94%)	46% (-46-87%)	NE	NE	
**Overall**	77% (37–92%)*	47% (22–64%^)^*	20% (-4-38%)	64% (50–74%)*	59% (30–76%)*	

*Indicates P < 0·05

IVE: Influenza vaccine effectiveness; CI: confidence interval; NE: Not estimable due to the low number of cases or vaccinated cases.

#### Unadjusted IVE against influenza A(H1N1)

The IVE against former seasonal A(H1N1) could only be estimated for the 2004/05 and 2007/08 influenza seasons due to absence or low number of cases in the other seasons. The overall IVE against influenza A(H1N1) for these two seasons was 77% (95% CI 37 to 92%). For influenza season 2007/08 IVE against A(H1N1) was statistically significant; although the vaccine did not match the circulating subtype (Table 2).

#### Unadjusted IVE against influenza A(H1N1)pdm09

The overall IVE against influenza A(H1N1)pdm09 was 47% (95% CI 22 to 64%). The seasonal IVE for the pandemic season (2009/10) for the emerged influenza virus A(H1N1)pdm09, was 25% (95% CI -72 to 68%) though not statistically significant. For the seasons thereafter, the seasonal IVE increased to 43% (95% CI -3 to 68%) in 2010/11, 51% (95% CI 3 to 75%) in 2012/13, which was statistically significant, and 72% (95% CI -12 to 94%) in 2013/14 (Table 2).

#### Unadjusted IVE against influenza B

The overall IVE against influenza B (total of B Yamagata and B Victoria) was 64% (95% CI 50 to 74%), which was statistically significant. For the influenza seasons 2007/08, 2010/11 and 2012/13 IVE against influenza B was statistically significant (Table 2)

The IVE against influenza virus B Victoria could only be determined for 2010/11 influenza season which was statistically significant with 76% (95% CI 49 to 89%). Except for the influenza season 2012/13, only a limited number of influenza B Yamagata cases were detected in the influenza seasons 2010/11 through 2013/14. The overall IVE against influenza B Yamagata was 59% (95% CI 30 to 76%), which was statistically significant. The season-specific IVE against influenza B Yamagata was only significant for 2012/2013 (IVE: 67%; 95% CI 40 to 82%) (Table 2).

### Virological characteristics of influenza seasons

In Table 3, the proportion of influenza virus (sub)types/lineages based on the Netherlands National Influenza Center (NIC) data is presented. The NIC data comes from the Sentinel Practices of NIVEL Primary Care Database and hospital laboratories that submitted influenza virus isolates or clinical specimens with a specimen collection date in week 40 of one year through week 39 of the following year. Based on the NIC data, influenza vaccine strains matched or partially matched the circulating viruses in 2005/06, 2006/07, 2008/09, and 2010/11 influenza seasons (Table 3) [23–33]. Influenza A(H3N2) was predominant in six influenza seasons: 2003/04, 2004/05, 2005/06, 2006/07 and 2008/09 and 2011/12. Among these six seasons, four influenza seasons were characterised by the circulation of a drifted A(H3N2) strain [reference strains 2003/04: A/Fujian/411/02(H3N2), 2004/05: A/California/7/04(H3N2), 2005/06:A/Wisconsin/67/05(H3N2), and 2011/12: A/Victoria/361/11(H3N2)]; and in three seasons (i.e. 2005/06, 2006/07, and 2008/09) influenza vaccine matched the A(H3N2) virus subtype.

**Table 3:** Proportion of virus (sub)types/lineages (%) and vaccine mismatch per subtype/lineage based on virus isolates and specimens submitted to the NIC in week 40 of one year through week 39 of the following year, seasons 2003/04 through 2013/14, Netherlands.

**Influenza season**											
**Virus (sub)type**	**2003/04**	**2004/05**	**2005/06**	**2006/07**	**2007/08**	**2008/09**	**2009/2010**	**2010/11**	**2011/12**	**2012/13**	**2013/2014**
**Proportion of virus (sub) types (%)**											
**A**	99	80	64	99	49	92	100	60	90	69	94
**A(H1N1)^1^**	0	18	4	13	86	1	100	97	1	39	40
**A(H3N2)**	100	82	96	87	14	99	0	3	99	61	60
**B**	1	20	36	1	51	8	0	40	10	31	6
**B**	Y	Y	Y/V	Y	Y	V	NA	Y/V	Y/V	Y/V	Y/V
**B/Vic**			91					95	12	6	24
**B/Yam**			9					5	88	94	76
**Mismatch per subtype**											
**A(H3N2)**	Yes	Yes	No	No	No	No	NA	No	Yes	Yes	Yes
**A(H1N1)^1^**	NA	No	No	Yes	Yes	No	Yes^2^	No	No	No	No
**B/Vic**	NA	NA	Yes (Yam in vaccine)	NA	NA	Yes (Yam in vaccine)	NA	No	No	NA	NA
**B/Yam**	Yes (Vic in vaccine)	Yes (Antigenic mismatch)	Yes (Antigenic mismatch)	Yes(Vic in vaccine)	Yes (Vic in vaccine)	NA	NA	Yes (Antigenic mismatch)	Yes (Vic in vaccine)	Yes (Antigenic mismatch)	Yes (Antigenic mismatch)
**Vaccine mismatch**	Mismatch	Mismatch	Partially match	Partially match	Mismatch	Match	Mismatch	Match	Mismatch	Mismatch	Mismatch

V: B/Victoria/2/87-lineage; Y: B/Yamagata/16/88-lineage; NA: Not applicable.

^1^2003/2004 through 2008/2009 season former seasonal A(H1N1); 2009/2010 through 2013/2014 season A(H1N1)pdm09.

^2^Seasonal A(H1N1) was used in the vaccine; A(H1N1)pdm09 monovalent vaccine was available late in the 2009/10 influenza season.

In general the activity of the former seasonal A(H1N1) influenza subtype was low and A(H1N1) or A(H1N1)pdm09 mainly co-circulated with influenza B virus (2007/08, 2012/13, 2013/14) (Table 2). In 2009/10 influenza season, the pandemic influenza virus A(H1N1)pdm09 emerged which was antigenically different from the seasonal influenza vaccine [i.e. circulating virus A/California/7/2009(H1N1)pdm09 vs. vaccine strain A/Brisbane/59/2007(H1N1)].

In five influenza seasons, both influenza B/Yamagata and B/Victoria lineages were circulating; in three (2011/12, 2012/13 and 2013/14) the proportion of influenza B/Yamagata lineage was higher (i.e. > 60%). In six seasons (2003/04, 2005/06, 2006/07, 2007/08, 2008/09 and 2011/12) circulating influenza B virus belonged to the opposite lineage of the vaccine.

**Fig 2:**
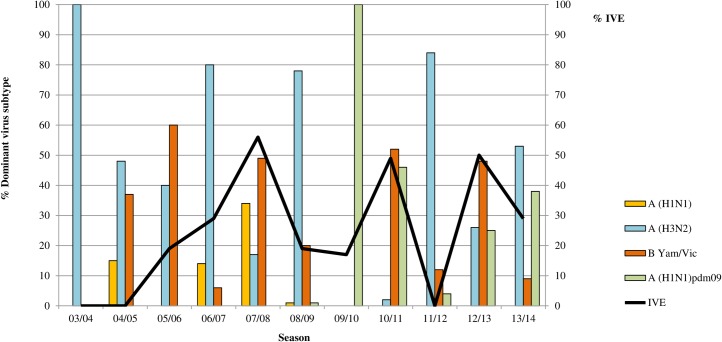
IVE for the seasons 2003/04-2013/14 and distribution of circulating influenza virus (sub)types according to data from Sentinel Practices of NIVEL Primary Care Database. IVE: influenza vaccine effectiveness. IVE estimates are relatively low (i.e. <30%), during influenza seasons with H3N2 as the predominant virus subtype (i.e. 2003/04, 2004/05, 2006/07, 2008/09, 2011/12, and 2013/14).

Fig 2 presents the distribution of circulating influenza virus (sub)types according to data from the Sentinel Practices of NIVEL Primary Care Database for each of the seasons. Remarkebly, in the six seasons in which the IVE was low (i.e. < 30%), the proportion of influenza virus A(H3N2) virus was high (i.e. 48–100%), while in the three seasons in which the IVE was moderate to high (i.e. > 50%), the proportion of influenza virus A(H3N2) was much lower (i.e. 2–26%).

In general the activity of the former seasonal A(H1N1) influenza subtype was low and A(H1N1) or A(H1N1)pdm09 mainly co-circulated with influenza B virus (2007/08, 2012/13, 2013/14) (Table 2). In 2009/10 influenza season, the pandemic influenza virus A(H1N1)pdm09 emerged which was antigenically different from the seasonal influenza vaccine.

In five influenza seasons, both influenza B/Yamagata and B/Victoria lineages were circulating; in three (2011/12, 2012/13 and 2013/14) the proportion of influenza B/Yamagata lineage was higher (i.e. > 60%). In six seasons (2003/04, 2005/06, 2006/07, 2007/08, 2008/09 and 2011/12) circulating influenza B virus belonged to the opposite lineage of the vaccine.

Finally, considering the distribution of circulating influenza virueses during each influenza season, the vaccine showed lower effectiveness (i.e. < 30%) during influenza seasons with relatively high influenza A(H3N2) virus activity (i.e. the proportion of A(H3N2) virus ranged from 48% to 100%) (2003/04, 2004/05, 2006/07, 2008/09, 2011/12, 2013/14), while in the three seasons in which the IVE was moderate to high (i.e. > 50%) (i.e. 2007/08, 2010/11, 2012/13), the proportion of influenza virus A(H3N2) was much lower (i.e. 2–26%).

## Discussion

In the present study, seasonal influenza vaccine strains did not match perfectly the circulating viruses in many influenza seasons (seven vs. four seasons). However, influenza vaccine showed moderate effectiveness when vaccine (partially) matched circulating viruses (four seasons) (IVE: 40%; 95% CI 18 to 56%) and low effectiveness when vaccine did not match (seven seasons) (IVE: 20%; 95% CI -5 to 38%). Moreover, the overall IVE as well as the (sub)type/lineage specific IVE showed extreme variability from season to season.

The estimated IVE for former A(H1N1) for the two influenza seasons 2004/05 and 2007/08 appeared to be consistently high (> 65%). The high IVE for influenza A(H1N1)pmd09 for the seasons 2010/11 onwards, corresponded well with the virological data that showed no drift variants and no vaccine mismatch for this subtype. The vaccine did not show statistically significant effectiveness against influenza virus B (total) for influenza seasons 2004/05, 2005/06, 2008/09 and 2011/12 that could be explained by vaccine mismatch with the circulating influenza B virus. The high IVE for influenza virus B Victoria lineage in influenza seasons 2010/11 also corresponded well with the absence of a vaccine mismatch for this lineage. On the other hand, the high IVE for influenza B virus (total) in 2007/08 (when Victoria lineage was in the vaccine) and for influenza virus B Yamagata lineage in 2012/13 (when there was an antigenic mismatch and the majority of the reported B/Yamagata/16/88 viruses were antigenically more closely related to B/Massachusetts/2/2012-like) [31] contradicted the fact that in those seasons vaccine did not match the circulating influenza B virus. Despite that, as discussed by a similar Canadian study, the high IVE for influenza B virus in 2007/08 influenza season could partly be explained by the possibility of cross-protection between the lineages [33].

Overall, IVE was low for most of the seasons in which influenza virus A(H3N2) dominated. Moreover, there was an inconsistency between the IVE estimates and vaccine match status for several influenza seasons. In two out of three influenza seasons (2005/06 and 2007/08) in which vaccine did not show effectiveness against influenza A(H3N2), vaccine strain matched the circulating virus subtype. Additionally, in three out of six seasons in which some degree of vaccine effectiveness was found (2003/04, 2011/12 and 2013/14) influenza vaccine strain mismatched the circulating influenza virus A(H3N2) and in two seasons (2003/04 and 2013/14) there was an antigenic drift compared to the previous influenza seasons.

Similarly, other studies in Europe, Canada and the USA have also observed a lower IVE for the influenza subtype A(H3N2), although vaccine matched the circulating virus subtype [10,34–36].

The inconsistency between the vaccine antigenic match and vaccine effectiveness is complex and has been reported in both observational studies and clinical trials [37,38]. Virological as well as epidemiological factors could contribute to this unexplained inconsistency. From the virological perspective, a recently conducted study has shown that ferret post-infection antisera responds differently than human post-vaccination sera to influenza A(H3N2) virus [39]. This finding indicates that new techniques such as human serologic testing should be used to improve vaccine virus strains selection. From the epidemiological perspective, it has been shown that the global circulation of influenza virus types and subtypes differs substantially [40]. For instance, influenza A(H3N2) has a faster antigenic evolution rate, occur more frequently, and result in more infection among adult population [40]. Hence, these epidemiological factors could result in different proportion of cases in each influenza season and consequently affect the IVE [5].

This is the first in depth multiple season virus type and subtype specific test-negative case-control study conducted among the general population in the Netherlands. Data provided by the Sentinel Practices of NIVEL Primary Care Database and laboratory diagnoses allowed estimating IVE over 11 influenza seasons (2003/04 through 2013/14). One of the strengths of this study is using datasets over a large number of influenza seasons which enabled us to not only assess the overall and season specific IVE, but also to study the potential association between the influenza virus types and subtypes/lineages.

Though, in order to interpret the results, it is important to note the study limitations. Firstly, due to the observational nature of the study design, it is unlikely that included ILI and ARI cases in the current study are a random sample of all individuals with ILI and ARI in the population. Moreover, although it is expected that GPs select the ILI and ARI patients randomly; in reality collecting a swab from a patient may partly depend on the GP discretion and patients’ consent. Secondly, due to the low power especially in some influenza seasons, we either could not estimate the IVE within influenza types/subtypes or we found rather wide confidence intervals. Besides, due to the low power, the adjusted IVE for influenza subtypes could not be calculated and subgroup analysis of VE based on baseline characteristics of the patients (e.g. age and presence of chronic diseases) for each influenza season could not be performed. Thirdly, the distribution of the ILI and ARI patients as well as the health care-seeking behavior of the two patient groups may differ in the influenza season, which could consequently lead to misclassification bias. However, since we did not find any confounding effect for the diagnosis variable (ILI and ARI), it is unlikely that it could influence our estimates. Finally, in the current study in order to estimate IVE, test-negative design case-control study as the most accurate observational study design has been used [35,41]. However, similar to other observational study designs, test-negative design has methodological limitations, which could bias the IVE estimates. Although the design partially controls for the bias due to the similar health care-seeking behavior among cases and controls, it still lacks randomization of influenza vaccination. For example, elderly population and patients with chronic medical conditions are more likely to get vaccinated due to the higher risk of developing severe influenza complications. As a result, patients among these high-risk groups could be more frequently selected for influenza virus infection diagnosis [42].

## Conclusions

In conclusion, the Sentinel Practices of NIVEL Primary Care database provides a suitable data source to measure IVE in the Netherlands. Based on our results, influenza vaccine had the highest protective effect against A(H1N1), A(H1N1)pdm09 and B influenza viruses. The overall IVE was particularly low during the seasons with A(H3N2) as the predominant influenza virus subtype. Moreover, vaccine showed moderate effectiveness when matched and low effectiveness when mismatched the circulating viruses. Therefore, efforts should be renewed to improve vaccine production procedures and to explore the potential factors that especially influence IVE against different virus subtypes in each influenza season such as age and presence of chronic medical conditions.

## Supporting Information

S1 File Statistical analysis. (DOCX)

S2 File Data sharing policies. (PDF)

S3 File Data transfer agreement. (DOCX)
