# Supplementary material for: Influenza Vaccine Effectiveness in the Netherlands from 2003/2004 through 2013/2014: The Importance of Circulating Influenza Virus Types and Subtypes
Source: PLoS One. 2017 Jan 9;12(1):e0169528. doi: 10.1371/journal.pone.0169528 (PMC5222508; doi:10.1371/journal.pone.0169528)
Supplement: S1 File — (DOCX) [file pone.0169528.s001.docx]

**S1 File.**

**STATISTICAL ANALYSIS**

Information on several patients characteristics including age, gender, presence of chronic medical condition, presence of respiratory allergy, diagnosis and interval between symptoms onset and swab collection were collected. In next step, variables associated with both, the outcome and the main exposure at alpha level of 5%, were included in the adjusted multiple logistic regression analysis and generalized linear mixed-effect model (GLMM). Since age category, presence of chronic medical conditions, and influenza season were the only variables associated with both outcome and exposure (potential confounders), only these three variables were included in the final model.

Seasonal influenza vaccine effectiveness (SIVE) and its 95% confidence interval (95% CI) was estimated by using the formula (1 − odds ratio) × 100%, where odds ratio is the ratio between the odds that an outcome will occur among the vaccinated, compared to the odds of the outcome occurring among the not vaccinated.

**SAS codes for GLMM to estimate adjusted IVE stratified by vaccine match status**

**proc** **Glimmix** data=TND_case_control order=data METHOD=QUAD(QPOINTS=**20**);

class agecat chron vaccine season match;

model INFLUENZA (event='1') = agecat chron vaccine

/dist=binomial link=logit or solution;

random intercept / subject=season;

**agecat:** age category 0-4, 5-14, 15-59 and ≥ 60 years,

**chron:** presence of chronic medical conditions (yes/no),

**vaccine:** vaccination status (yes/no),

**match:** vaccine match to the circulating viruses (yes/no),

**season:** influenza seasons.
