# Supplementary material for: Influenza Vaccine Effectiveness in the Netherlands from 2003/2004 through 2013/2014: The Importance of Circulating Influenza Virus Types and Subtypes
Source: PLoS One. 2017 Jan 9;12(1):e0169528. doi: 10.1371/journal.pone.0169528 (PMC5222508; doi:10.1371/journal.pone.0169528)
Supplement: S2 File — (PDF) [file pone.0169528.s002.pdf]

National Institute for Public Health
and the Environment
Ministry of Health, Welfare and Sport

Data Transfer Agreement

A. van Leeuwenhoeklaan 9
3721 MA Bilthoven
The Netherlands
P.O. Box 1
3720 BA Bilthoven
The Netherlands
www.rivm.nl

CoC Utrecht 30276683

T +31 30 274 91 11
F +31 30 274 29 71
info@rivm.nl

Our reference
DTA-2014-01

Disclosing Party:
The National Institute for Public Health and the Environment of The Netherlands, Center for Infectious Disease Control (CIb), Centre for Infectious Disease Research, Diagnostics and Screening (IDS), (further: RIVM)
Represented by Dr. Adam Meijer

Recipients:

ICON Clinical Research UK Ltd
6 Stoney Croft Rise, Chandlers Ford
Eastleigh, United Kingdom SO53 3LD
Represented by **Dr. Richard Pitman**

University of Groningen
Represented by **Prof. Dr. Maarten Postma**

I. RIVM and Recipient agree, for the benefit of public health, on collaboration for the realization of the Project "*Epidemiological impact and cost effectiveness of influenza-vaccination of children in the Netherlands*" (Further: The project).
Recipient will receive virologic and linked demographic, clinical and epidemiologic data required for the Project (further: 'Transferred Data') collected by RIVM through the NIVEL General Practitioner Surveillance.

II. RIVM, as Disclosing Party, grants Recipient a non-exclusive right to use the Transferred Data for the realization of the Project.

III. Upon receiving and accepting the Transferred Data, Recipient agrees to and undersigns the following conditions and obligations:

1. Recipient will not use Transferred Data otherwise than for the Project and purpose for which they were disclosed;

2. Recipient will not disclose Transferred Data to any third party without the prior written consent by the Disclosing Party;

3. Recipient ensures that internal distribution of Transferred Data shall take place on a strict need-to-know basis;

4. Recipient shall return to the Disclosing Party on demand all Transferred **Data which has been supplied to or acquired by the Recipient including all copies thereof and to delete all information stored in a machine readable form. If needed for the recording of ongoing obligations, the Recipient may however request to keep a copy for archival purposes only;**

Date
17 July 2014

Our reference
DTA-2014-01

5. Recipient will include at least one co-author of RIVM in any publication on the basis of the Transferred Data in national and international journals, whilst adhering to the ICMJE recommendations for authorship.

IV. Recipient shall be responsible for the fulfilment of the above obligations on the part of their employees and shall ensure that their employees remain so obliged, as far as legally possible, during and after the end of the Project and/or after the termination of employment.

The above shall not apply for disclosure or use of Transferred Data, if and in so far as the Recipient can show that:

- the Transferred Data become publicly available by means other than a breach of the Recipient's confidentiality obligations;
- the Disclosing Party subsequently informs the Recipient that the Transferred data are no longer confidential;
- Recipient is required to disclose Transferred Data in order to comply with applicable laws or regulations or with a court or administrative order.

V. The Recipient shall apply the same degree of care with regard to the Transferred Data disclosed within the scope of the Project as with its own confidential and/or proprietary information, but in no case less than reasonable care.

VI. Each Party shall promptly advise the other Party in writing of any unauthorized disclosure, misappropriation or misuse by any person of Transferred Data as soon as practicable after it becomes aware of such unauthorized disclosure, misappropriation or misuse.

Date: 17/7/2014

On behalf of Recipients:
ICON Clinical Research UK Ltd

[signature]

Dr. Richard Pitman

University of Groningen 18/7/'14

[signature]

Prof. Dr. Maarten Postma

Date: 17 July 2014

On behalf of Disclosing Party
RIVM-CIb / IDS, Bilthoven NL

[signature]

Dr. A. Meijer

University Groningen
8/1/2014
[signature], prof dr E. Hak

Date
8 January 2015

Our reference
DTA-2014-01

Extension to DTA-2014-01

1) The data provided to Recipient as described in DTA-2014-01 are allowed to be used by Prof. Dr. Eelko Hak, University of Groningen, and the co-workers under his direct responsibility, for influenza vaccine effectiveness studies.

2) In addition, for the purpose of these influenza effectiveness studies, RIVM provides additional metadata to Recipient.

3) All data described above are allowed to be used for influenza vaccine effectiveness studies by Recipient under the same conditions as described in DTA-2014-01.
