# Supplementary material for: Influenza Vaccine Effectiveness in the Netherlands from 2003/2004 through 2013/2014: The Importance of Circulating Influenza Virus Types and Subtypes
Source: PLoS One. 2017 Jan 9;12(1):e0169528. doi: 10.1371/journal.pone.0169528 (PMC5222508; doi:10.1371/journal.pone.0169528)
Supplement: S3 File — (DOCX) [file pone.0169528.s003.docx]

**S2 File.**

**Data sharing policies:**

1) We ask that you please explain the ethical or legal restriction preventing your data sharing. 

The data for the study was shared under a data transfer agreement restricting onward transfer of the data to third parties without prior agreement with the National Insitute of Public Health and the Environment. See the Data Transfer Agreement. However, researchers that want to use the data can contact dr. A. Meijer (adam.meijer@rivm.nl) for collaboration opportunities. 

2) Please provide the names of the parties involved in the data transfer agreement. 

National Institute for Public Health and the Environment, Bilthoven, the Netherlands, ICON Clinical Research UK Ltd, Eastleigh, United Kingdom and University of Groningen, Groningen, the Netherlands. See the  Data Transfer Agreement. 

3) Please provide the contact information (name, title, email address) to where data requests may be sent if the data is ethically or legally restricted. 

Researchers that want to use the data can contact dr. A. Meijer (adam.meijer@rivm.nl) for collaboration opportunities under similar conditions as outlined in the Data Transfer Agreement between National Institute for Public Health, ICON Clinical Research UK Ltd and the Environment and University Groningen.
